# Book Reviews

**Published:** 1868-12

**Authors:** 


### BIBLIOGRAPHICAL NOTICES.

*A Practical Treatise on Mechanical Dentistry.*—By Joseph Richardson, D.D.S., M.D. Second Edition. Publishers, Lindsay & Blakiston, Philadelphia.

For a number of years the first edition of this work has been familiar to the dental practitioner, and it is with pleasure that we note the issue of a second edition, very much enlarged as regards both the text and illustrations.

Dr Richardson deserves the thanks of the profession for the able manner in which he has described the various manipulations of the laboratory, and has succeeded in making his treatise a practical guide for the practitioner as well as for the student.

An examination of the new edition shows us that the chapter on "Vulcanite Base" has been considerably extended, and all the valuable improvements made in this process fully described.

In the chapter on "Continuous Gum Work," some eight pages are devoted to a description by Dr. Jno. Allen, (who has no superior in a process which he has made a specialty) of the manipulations at present practiced by him in the construction of this work.

In the chapter on "Defects of the Palatal Organs and their Treatment by Artificial Means," we find a full description of the "Kingsley Artificial Velum and Palate, which was written by Dr. Kingsley at the request of the author, and embraces all the improvements made up to the present time in this branch of the dental art. These clear and concise accounts of important processes by Drs. Allen and Kingsley are of themselves sufficient to render the work one of value and interest to all engaged in the practice of dentistry.

A chapter on Aluminium, in which are described the different modes of working this metal, has been added to the new edition. Among the processes described is that of Dr. Bean's "Cast Aluminium Base;" also Dr. Franklin's method of attaching a rim with solder to a swaged aluminium plate, together with a formula of Dr. Starr's solder for aluminium.

The additions made to this treatise on Mechanical Dentistry, will render it a valuable text book. The mechanical execution of the work is all that could be desired.

---

*The Baltimore Medical Bulletin* is the title of a new journal edited by Prof. Edward Warren of the Washington University. The want of a medical organ in Baltimore has long been felt, and we trust its success may be such that it will soon cease to be an experiment.

The high reputation of the editor in his profession, as a practitioner and a lecturer, is a sufficient guarantee as to the manner in which this new candidate for professional favor will be conducted; and the very moderate subscription price $2. per year, places it within the reach of all.

It is issued on the 1st and 15th of each month.

---

*The Medical Gazette.*—As another acknowledgement of dentistry as a specialty of general medicine and surgery, the *Medical Gazette* has added a department of Dental Science.

We announce this addition with pleasure, as the judicious selections it contains, taken from dental journals, renders the *Gazette* a valuable medical journal for the dental, as it has hitherto been for the medical practitioner. We consider the *Medical Gazette* one of the best of our exchanges, and trust that this improvement may greatly increase its circulation among dentists.

### EDITORIAL DEPARTMENT.

